# A strategy to build and validate a prognostic biomarker model based on RT-qPCR gene expression and clinical covariates

**DOI:** 10.1186/s12859-015-0537-9

**Published:** 2015-03-28

**Authors:** Maud Tournoud, Audrey Larue, Marie-Angelique Cazalis, Fabienne Venet, Alexandre Pachot, Guillaume Monneret, Alain Lepape, Jean-Baptiste Veyrieras

**Affiliations:** 1 Bioinformatics Research Department, bioMérieux, Marcy L’Etoile France; 2 Medical Diagnostic Discovery Department, bioMérieux, Marcy L’Etoile France; 3 Laboratoire Commun de Recherche, Hospices Civils de Lyon, Lyon, France

**Keywords:** Prognostic survival model, RT-qPCR gene expression measurement, Cross-validation, Performance estimation, Model optimism

## Abstract

**Background:**

Construction and validation of a prognostic model for survival data in the clinical domain is still an active field of research. Nevertheless there is no consensus on how to develop routine prognostic tests based on a combination of RT-qPCR biomarkers and clinical or demographic variables. In particular, the estimation of the model performance requires to properly account for the RT-qPCR experimental design.

**Results:**

We present a strategy to build, select, and validate a prognostic model for survival data based on a combination of RT-qPCR biomarkers and clinical or demographic data and we provide an illustration on a real clinical dataset. First, we compare two cross-validation schemes: a classical outcome-stratified cross-validation scheme and an alternative one that accounts for the RT-qPCR plate design, especially when samples are processed by batches. The latter is intended to limit the performance discrepancies, also called the validation surprise, between the training and the test sets. Second, strategies for model building (covariate selection, functional relationship modeling, and statistical model) as well as performance indicators estimation are presented. Since in practice several prognostic models can exhibit similar performances, complementary criteria for model selection are discussed: the stability of the selected variables, the model optimism, and the impact of the omitted variables on the model performance.

**Conclusion:**

On the training dataset, appropriate resampling methods are expected to prevent from any upward biases due to unaccounted technical and biological variability that may arise from the experimental and intrinsic design of the RT-qPCR assay. Moreover, the stability of the selected variables, the model optimism, and the impact of the omitted variables on the model performances are pivotal indicators to select the optimal model to be validated on the test dataset.

**Electronic supplementary material:**

The online version of this article (doi:10.1186/s12859-015-0537-9) contains supplementary material, which is available to authorized users.

## Background

Prognostic survival models refer to the “quantification of the survival prognosis of patients based on information at start of follow-up (*t*=0)” [1]. Here, survival should be taken in the broadest sense and relates to the probability of an individual to develop a given outcome over a specific time. Frequently studied outcomes are the time-to-death or the time-to-disease progression.

The development of a prognostic model usually involves two main steps: model building and validation. The prognostic model is built based on a training or learning sample set after which the model is validated using test or validation samples. The quality of a prognostic model is measured through its performance, i.e. the ability of the model to correctly predict the prognosis of a patient based on his observed predictors.

Prognostic model building and validation is an active field of research in biostatistics: for instance, Steyerberg [2] presented several successful applications for public health, clinical practice and medical research. Although many prognostic models have been published in the medical literature every year, such models are rarely implemented in clinical practice. This is mainly due to the lack of generalization of these models, meaning that the performance of the reported models is not as good on new independent datasets as they were on the dataset used to build the model [3]. The three most frequent reasons for this lack of generalization are: *i)* deficiencies in the design or in the modeling methods used to derive the model *ii)* upward biases regarding the estimation of the performance, and *iii)* differences between training and test samples, including differences in health-care systems, methods of covariate measurements, and patient characteristics [4-8].

The objective of this article is to present a strategy for prognostic survival model building, selection, and validation for the development of routine prognostic tests based on a combination of RT-qPCR biomarkers and clinical or demographic variables and to illustrate it on a real dataset. We will focus on three main issues: resampling strategy, performance indicator estimation, and criteria for model selection.

First, accurate estimation of model performance is a crucial step in the model validation process. Performance estimated on the training samples used for model building, i.e. apparent performance, is very likely to be upwardly biased. Resampling methods, such as *k*-fold cross-validation and bootstrap [9] are the most common techniques to estimate the actual performance. Resampling methods should properly capture the intrinsic technical variability of the biomarker assay. This is particularly important in RT-qPCR assays or any assay that processes samples batchwise, where a batch is defined to be a set of samples that undergo the same experimental conditions. In this article, we compare two cross-validation strategies; one method accounts for the technical and/or the biological/clinical variability related to the batch design of the biomarker study, while the other does not.

Second, two methods can be used to estimate the cross-validated performance indicators: the pooling and the averaging methods [10,11]. The pooling method estimates the performance at the end of a cross-validation repetition whereas the averaging method estimates the performance within each fold, and performances are then averaged over folds. The pooling method generally results in a smaller variance than the averaging method, which might be critical for small sample size or limited number of folds. However, depending on the cross-validation strategy, and the performance indicator considered, the pooling method can lead to biased estimations [10]. The advantage of both methods in the context of censored survival data will be discussed hereafter.

Third, selecting the optimal model is generally a difficult decision-making step. Indeed, several models might present similar performances. In this case, model performance may not be the sole criterium to select the optimal model. Complementary criteria for model selection will be discussed.

Finally, we will detail the construction of the selected model, and the subsequent validation steps; the complete strategy is then illustrated on a real dataset: the prognostic of mortality for septic shock patients in intensive care units.

## Methods

### Study design

#### Research protocol

According to the Helskinki declaration [12], the research protocol must be submitted for consideration, and approval to an independent ethics committee before the study begins. The work presented below belongs to a global study on ICU-induced immune dysfunctions. It has been approved by a local Institutional Review Board for ethics (Comité de Protection des Personnes Sud-Est II) which waived the need for informed consent. Indeed, this study was observational and biomarkers expressions were measured on residual blood after completion of routine follow-up (IRB#11263). This study was also registered at the French Ministry of Research and Teaching (#DC-2008-509) and recorded at the Commission Nationale de l’Informatique et des Libertés.

#### Sample size

The estimation of sample sizes for the training and test datasets is pivotal to drive patient inclusion and scale the corresponding efforts. Unfortunately, up to now there is no theoretical rational for training sample size estimation when *p*<*n* (i.e. the number of candidate predictors, namely *p*, is lower than the number of patients denoted *n*). A simulation study suggested that at least 10 events per candidate predictor (EPV) seems “prudent” [13], whereas Vittinghoff et al. [14] found bias and coverage problems with 5-9 EPV. The limitation of this study is that they focused on one primary predictor and they treated other variables as adjustment variables, i.e. they did not study the bias and coverage of adjustment covariates. Recently, Dobbin and Song [15] developed a method for sample size estimation to train a survival risk predictor in high-dimensional settings. The sample size is defined such that performance is within a given interval of the optimal. Application of the method to low-dimensional setting has not been evaluated by the authors and such an evaluation was out of the scope of our study. Regarding the sample size estimation of the test dataset, we propose to rely on the method devised by Shoenfeld for clinical trials [16]. With the final objective to validate a two-groups prognostic index, the approach can be adapted to test the null hypothesis of a death hazard ratio equal to one between the two prognostic groups (instead of the two arms of a clinical trial). Following Schoenfeld, the number of events required to test the null hypothesis *H*_0_:*Δ*=1, under the alternative hypothesis *H*_1_:*Δ*=*Δ*_0_, with a significance level of *α* and the power *β* is:

$$\frac{(z_{\beta} + z_{1-\alpha})^{2}}{p_{A} p_{B} (\ln(\Delta_{0}))^{2}} $$

with *z*_1−*α*_ and *z*_*β*_ the 1−*α* and *β* percentiles of the Normal distribution, *Δ*_0_ the hazard ratio for the good vs. the bad prognostic group under the alternative hypothesis, and *p*_*A*_ and *p*_*B*_ the proportion of patients in the 2 prognostic groups.

#### RT-qPCR experimental design

The real-time, fluorescence-based Reverse Transcription quantitative Polymerase Chain Reaction (RT-qPCR) is widely used to measure the expression levels of genes [17]. This technology relies on two main steps: the reverse transcription (RT) of messenger RNA (mRNA) into complementary DNA (cDNA) and the quantification of cDNA using real-time PCR. In the RT step, samples are processed by batch: several samples are processed at the same time in the same RT run. Moreover, PCR measurements are performed on PCR plates with a variable number of wells. Ideally, a batch should be defined so that all the samples within a batch will be subject to the same experimental conditions from the mRNA extraction to the final amplification measurements. This can be done by gathering samples based on the technical factors of the entire analytical chain: sample preparation, RT, and PCR steps. In particular, samples processed in the same RT run and with genes expression measured on the same PCR plate should belong to the same batch. It is also important to control for balanced patient’s outcomes across batches by including both survivor and non-survivor patients within each batch, when studying time-to-death event data. Also, demographic/clinical variables that are expected to be both associated with gene expression variables and outcome should be balanced across batches or included into the model for statistical adjustment.

Although, RT-PCR is a broadly used and standardized method, it is still important to pay particular attention to the control of the technical variability of the measurements. First, the technical variability can be decreased thanks to normalization methods based on relative quantification. In particular, relative quantifications can be derived with respect to reference or housekeeping genes selected for their stable expression across samples [18]. Second, maximizing the number of samples (instead of maximizing the number of genes) per PCR plate is recommended to favor the comparison of gene expression across patients [18]. Ideally, this would allow us to measure only one candidate biomarker at a time within a PCR plate.

Finally, we recommend that training and test datasets are acquired in a row, while processing training and test samples in different PCR batches, so that the technical/biological variability between the two datasets is not larger than the technical/biological variability within the training dataset.

### Resampling strategy

Resampling methods allow us to mimic the validation step within the training dataset only, in order to select models with good generalization performance. *N*−times *k*−fold cross-validation is a standard resampling method used to estimate model performance on the training dataset. Briefly, for each of the *N* repetitions, the training dataset is split into two parts: the training-fold and the test-fold dataset. (Please refer to the discussion for the rational for the choice of cross validation versus bootstrap as a resampling strategy in this context). The training-fold dataset includes (*k*−1)/*k* of the training dataset patients and the test-fold dataset includes the remaining 1/*k* of the training dataset patients. The procedure is repeated *k* times so that all of the patients are included once in a test-fold dataset. Each time, the training-fold dataset is used to build the prognostic model (including data-preprocessing and normalization, variable selection and the estimations of the model parameters) and to estimate its apparent performance; then the corresponding test-fold dataset is used for (cross)-validated performance estimation. The apparent performance estimated on the training-fold dataset is expected to be overly-optimistic because the training-fold dataset is both used to build the model and estimate its performance. The difference between the apparent and the validated performance corresponds to the model optimism. The more complex a model is, the larger the optimism is expected to be.

Moreover, resampling methods should properly capture the intrinsic technical variability of the biomarker assay. If the technical variability between the training and the test datasets is larger than the technical variability between the training samples, the performance estimated on the training samples is likely to be optimistic. The difference between the estimation of the performance in the training and the test datasets is called the “validation surprise” and reflects the lack of generalization of the model. This problem may happen with RT-qPCR assays or any assay that processes samples by batch. Hence, the within batch variability is likely to be smaller than the between batch variability. As stated in the introduction, we compared two resampling strategies: strategy A and B. Strategy A is a stratified cross-validation strategy where test-fold samples are randomly sampled across batches so that the proportion of events is the same in each fold. Contrary to strategy A, strategy B tries to capture the technical and biological variability between the training and test datasets where entire PCR batches are randomly sampled and corresponding patients are set apart for test-fold data. This strategy does not ensure that the proportion of events is the same in each fold, except if the batches were designed to have the same proportion of events. With resampling strategy B, the variability between the training and the test fold datasets is expected to mimic the variability between the training and the test datasets. By doing so, cross-validated performance should better capture the between dataset variability with a lower risk of observing a significant validation surprise.

### Model building strategy

A model building strategy is defined by: i) a selection method providing the eligible covariates to enter into the model, ii) the functional relationship between each selected continuous covariate and the outcome, iii) a survival model, and iv) an optional penalization method to shrink the model coefficients. Among the available selection methods, we could cite: ad-hoc inclusion of a variable in the model, sequential strategies (e.g. backward elimination) using the Akaike Information Criterion (AIC) as selection criterion [19], or L1-penalized approach: the Cox-lasso model [20], the Cox-adaptive lasso model [21], and the Cox-SCAD model [22] which performs covariate selection and estimation at the same time. For L1-penalized approaches, variations of the L1 penalty parameters naturally lead to different model complexities. These penalized strategies can be used either as a pure variable selection strategy (meaning that the selected covariates are subsequently included in a classical Cox model), or as penalized models (meaning that coefficients of selected covariates are shrunken towards 0, the amount of shrinkage being controlled by the L1 penalty parameters). Regarding functional relationship modeling, Royston and Altman [23] developed a Multiple Fractional Polynomials (MFP) strategy which performs adequate description of the relationship and variable selection at the same time. Two parameters are needed to control for the degree of flexibility of the relationship and degree of complexity (number of included covariates) of the model. Again, variations of these two parameters leads to different strategies for model building. With respect to the survival model itself, the Cox model is very popular because of its simplicity, although the proportional HR assumption has to be checked a posteriori. Moreover, the standard Cox model assumes independent censoring, conditional to the covariates included in the model. Finally, a L2 (ridge) penalization step could be also considered in order to shrink the model coefficients by imposing a constraint on their effect size [24]. The shrunken coefficients then present a smaller variance. Please refer to Table 1 for a summary of the prognostic model building strategies.

**Table 1:** **Summary of strategies to build prognostic survival models**

**Strategy**	**Variable selection method**	**Functional relationship**	**Survival model**	**Coefficients shrinkage**
Uni_Cox-[1-9]*	Each candidate covariate was selected apriori and included in a univariate model.There is 1 suffix number in the model name per selected covariate	All candidate covariate are continuous, and a linear relationship is assumed	Cox model	
bwAIC_FP_Cox-[1-5]* bwAIC_FP/C2-3Fac_Cox-[1-5]*	Backward elimination using AIC criterion	Fractional polynomial to model functionalrelationship. The suffix in the model namecorresponds to the degree of flexibility,controlled with the parameter *α*=0.05, 0.1,0.2,0.3,0.4. In the bwAIC_FP/C2-3Fac_Cox-[1-5] models, variable C2 and C3 are dichotomized according to expert knowledge. Larger a values correspond to more flexible relationships.	Cox model	
MFP_Cox-[1-15]*	MFP procedure for variable selection controlled by the parameter select =0.05,0.10, 0.15. Larger select values correspond to less stringent variable selection. Suffix in the model name corresponds to a combination of select and alpha values	Fractional polynomial to model functional relationship. The parameter *α* controls the degree of flexibility, *α*=0.05,0.1,0.2,0.3,0.4. Larger a values correspond to more flexible relationships	Cox model	
Lasso-[1-5]* Lasso_C2-3Fac-[1-5]	L1 penalty for variable selection, controlled by the parameter *λ*=0.01,0.1,1,10,100. Larger *λ* values correspond to sparser models. Suffix in the model name correspond to the level of penalization.	Linear relationship, except for C2 and C3 which have been dichotomized according to expert knowledge in the Lasso_C2-3Fac_Cox- models.	Lasso Cox model	L1 penalty
aLasso-[1-5]* aLasso_C2-3Fac-[1-5]*	L1 penalty for variable selection, controlled by the parameter *λ*=0.01,0.1,1,10,100. Larger *λ* values correspond to sparser models. Suffix in the model name correspond to the level of penalization.	Linear relationship, except for C2 and C3 which have been dichotomized according to expert knowledge in the aLasso_C2-3Fac_Cox- models.	Adaptive LassoCox model	Adaptive lasso penalty for coefficients shrinkage (larger coefficients are less shrinked towards 0 in the adaptive lasso model than in the lasso model.
SCAD-[1-5]* SCAD_C2-3Fac-[1-5]*	L1 penalty for variable selection, controlled by the parameter *λ*=0.01,0.1,1,10,100. Larger *λ* values correspond to sparser models. Suffix in the model name correspond to the level of penalization.	Linear relationship, except for C2 and C3 which have been dichotomized according to expert knowledge in the SCAD_Lasso_C2-3Fac_Cox- models.	SCAD Cox model	SCAD penalty for coefficients shrinkage (larger coefficients are less shrinked towards 0 in the SCAD model than in the lasso model.
Lasso_Cox-[1-5]* Lasso_C2-3Fac_Cox-[1-5]*	L1 penalty for variable selection, controlled by the parameter *λ*=0.01,0.1,1,10,100. Larger *λ* values correspond to sparser models. Suffix in the model name correspond to the level of penalization.	Linear relationship, except for C2 and C3 which have been dichotomized according to expert knowledge in the Lasso_C2-3Fac_Cox- models.	Cox model	

The first column gives the names of the tested strategies. The strategies cover a wide range of state-of-the-art methods from both low and high dimensional settings. The second column details the variable selection method used; the third column the functional relationship for continuous covariates; the fourth column the survival model; and the last column the coefficients shrinkage strategy if any. The ∗ suffix indicates the index of the prognostic model falling in the strategy. For example, Uni_Cox −[1−9]∗ means that 9 univariate Cox models were built, each of them being suffixed by digit 1 to 9.

### Prognostic model performance

Two main methods can be used to estimate performance indicators. First, the pooling method estimates the performance indicator at the end of the cross-validation process, i.e. when the predicted scores are available for all patients. Second, the averaging method estimates the performance indicator at the end of each fold. In this case, the *k* estimations of the performance indicator are averaged at the end of the cross-validation process. The main advantage of the pooling method over the averaging method is that the performance indicator is expected to present a smaller standard error [10]. However, depending on the resampling strategy and the prediction score used, the performance indicator estimation obtained with the pooling method could be biased downward because prediction scores cannot be directly compared from one fold to another. Performance indicators used in this study fall into three categories: overall performance measures (Brier score), discrimination measures (C-index, sensitivity, specificity, AUC) and calibration measures. Overall performance measures are based on the difference between predicted and observed outcomes and discrimination measures are related to the ability of the model to separate individuals who develop the outcome from those that do not. Calibration measures help to make sure that observed and predicted survival functions are comparable. Model calibration can be evaluated by comparing model predicted survival and Kaplan-Meier survival estimates at a fixed time point (see [25] for details).

#### Overall performance measure

The Brier score is a time-dependent measure of the prediction error [26]. The expected Brier score at time *t* is defined as $BS(t)=E\left [(I(T>t) - \hat {\pi }(t|Z))^{2}\right ]$ where *I*(*T* > *t*) = 1 if the survival time *T* > *t* and $\hat {\pi }(t|Z)$ is the estimated survival function given covariate vector *Z* (derived from the fitted model). For the Cox model, the Breslow [27] estimator of the baseline hazard was used to estimate the survival at time *t*. Graf et al. [26] provided an empirical estimation of the Brier score, in presence of censoring. The predicted survival for each test-fold sample $\hat {\pi }(t|Z)$, depends on the survival distribution in the corresponding training-fold sample. Hence, if the survival distributions are different between the training-fold and the test-fold data, the Brier score will be biased upward. This will typically be the case for unstratified cross-validation, or when using cross-validation strategy B when the outcome is unbalanced between batches. In practice, if the training and test-fold data outcome distributions are comparable, we recommend to use the pooling method to estimate the Brier score because it should lead to a lower standard error and no bias.

#### Discrimination measures

The C-index or concordance index is: *c*=*P*[*Z*_*j*_ *β*>*Z*_*i*_ *β*|*T*_*j*_<*T*_*i*_]. The C-index indicates the probability that subject *j* who died at an earlier time *T*_*j*_ than subject *i* has a larger Cox model linear predictor (*Z*_*j*_ *β*) than subject *i*. This is a time-independent performance indicator. In practice, linear predictors of the Cox model are comparable across test-folds and the pooling method could be used to estimate the C-index whatever the differences between training-fold baseline hazards, and hence whatever the resampling strategy used (see Additional file 1 for illustration with simulations).

Heagerty et al. [28] proposed a time-dependent estimator of the cumulative sensitivity and dynamic specificity for a continuous risk factor and censored survival data. In the following equations *M*_*i*_ is the continuous marker (or linear predictor of a Cox model in case of a multivariate model) for patient *i*,*T*_*i*_ the event time for patient *i*,*c* is a threshold value of this *M*_*i*_ marker or index, and *t* is a time point. The “cumulative/dynamic” definition of sensitivity/specificity is then:

$$\begin{array}{@{}rcl@{}} \text{sensitivity}(c;t)&=&P(M_{i}>c|T_{i} \leq t) \\ \text{specificity}(c;t)&=&P(M_{i} \leq c|T_{i} > t) \end{array} $$

Here, the sensitivity(*c*;*t*) measures the expected fraction of subjects with a marker greater than *c* among the subpopulation of individuals who die before time *t*, and the specificity(*c*;*t*) measures the expected fraction of subjects with a marker less than *c* among those who survive beyond *t*. The advantage of this definition is that at any time *t*, we have two groups, those who have already experienced the event (case group), and those who have not (control group). These performance indicators thus capture the discrimination level between individuals who have the event before time *t* and the individuals who survive beyond time *t* (especially when a particular value of *t* may present a scientific and/or clinical interest). In practice, as for the C-index, the linear predictors of the Cox model are comparable across test-folds and the pooling strategy could be used whatever the resampling strategy.

### Model selection

In practice, a large number of models need to be evaluated. Thus, small differences in cross-validated performances of the models have to be interpreted with caution. In order to narrow down the number of candidate models, we suggest a two-step model selection strategy. First, one needs to select a short-list of models with equally good performance and second apply additional model selection criteria to retain, ideally, a single model for the evaluation on the independent test dataset. The proposed additional selection criteria are: *i)* the stability of the selected variables across cross-validation repetitions, *ii)* the clinical interest in the selected variables, *iii)* the model optimism and *iv)* the impact of the omitted variables on the model performance.

#### Stability

The stability of the selected variables should be evaluated for automatic variable selection methods (e.g. backward elimination, MFP, L1-penalized Cox models, …); indeed, a selection method that selects more frequently the same set of covariates from the training-folds should be preferred, since such a selection method is less sensitive to the variability of the training dataset [29]. For selection methods that constrain the number of variables included into the prognostic model, the Kuncheva index [30] could be used to assess the model stability. The Kuncheva index is a stability measure between −1 and 1, with larger values for more stable models: *K* *I*(model−fold−i,model−fold−j)=(*r*−(*s* ^2^/*N*)/(*s*−(*s* ^2^/*N*)), with *s* the number of predictors, *r* the number of shared predictors between the training folds *i* and *j*, and *N* the number of candidate covariates. For selection methods without constraints on the number of variables included in the prognostic model, the frequency of inclusion of each covariate among the training-folds can be used as an indicator of stability.

#### Clinical interest

The clinical interest in the selected variables is a less objective criterion that would require opinion-driven inputs from biologists and clinicians (which are generally more difficult to leverage and describe statistically).

#### Model optimism

The model optimism [2] is defined as the difference between the actual performance (actual performance in the targeted population) minus the apparent performance (the estimated performance from the training dataset). Model optimism is a consequence of model overfitting. Thus, we propose estimating the so-called validation surprise as the difference between the apparent performance estimated directly on the training-fold data (also used for model building) and the performance estimated on the test-fold data. Besides, the level of overfitting depends on the strength of selected predictors [31], the dependence between predictors, and the complexity of the model. Indeed, if a model is too complex, it will over-fit the training data and not generalize well to new data. Hence, models with high degree of optimism should be avoided because they are expected to present less generalization power.

#### Impact of omitted covariates

Finally, some covariates could be excluded from the set of candidate covariates in case of limitations of the study sample size. The recommendation of 5-10 events per candidate covariate in the training dataset could lead to exclusion of some a priori less important variables from the set of candidate covariates. Hence, it is important to check i) whether some omitted covariates could still be associated with survival after adjustment on selected covariates, and ii) whether model performance could depend on the level of omitted covariates.

First, similar to Therneau and Grambsch [32], we propose to use the Martingale residuals to test the residual association between the survival and each omitted covariate. In case of no association between the Martingale residuals and the omitted covariate, the covariate was considered not associated with the survival. In practice, for each patient in the training-fold datasets, the Martingale residuals were averaged across all the folds and iterations.

Second, we suggest to test whether the sensitivity and the specificity depend on omitted covariates using the following logistic models (in absence of censoring):

$$\begin{array}{@{}rcl@{}} \text{logit}(P(M_{i}>c|T_{i} \leq t,Z_{i})) = Z_{i}\beta^{\text{sensi}}\\ \text{logit}(P(M_{i} \leq c|T_{i} > t,Z_{i})) = Z_{i}\beta^{\text{speci}} \end{array} $$

where, *M*_*i*_ is the linear predictor for patient *i* of the Cox model without the omitted covariate, *Z*_*i*_ is the omitted covariate for patient *i*, *β* ^sensi^ is the log odds ratio of the correct classification probability among deceased patients at time *t* for patients with covariate *Z*_*i*_=1 vs *Z*_*i*_=0, and *β* ^speci^ is the log odds ratio of the correct classification probability among survivor patients at time *t* for patients with covariate *Z*_*i*_=1 vs *Z*_*i*_=0. For example, if *β* ^sensi^>0, this means that the sensitivity is increased among patients with larger *Z* omitted covariate. Here, the vital status of each patient has to be known at each time point (i.e. there is no censoring). Under the assumption of independent censoring, we could restrict the previous estimation to patients with known vital status at time *t*, although the estimation of the impact of omitted covariates will have a larger variance compared to the case without censoring. (To compensate for the loss of information due to censoring, we could use inverse probability of censoring weighting or multiple imputations to impute censored failure times; this would however require further methodological development, which is beyond the scope of this paper.) In practice, it appears reasonable to restrict the evaluation to a given time-point *t*. Hence, for each omitted covariate, for each iteration, and each threshold *c*, two logistic models were built (one for sensitivity and one for specificity). This leads to a high number of models and tests for significant association between the omitted covariate and sensitivity/specificity. Given the large number of tests performed, we might expect several tests to show low p-values only by chance. Although p-values should be corrected for multiple testing, this additional criterion should not be considered as a formal statistical test but rather as an indicator of a potential association between performance indicators and each omitted covariate. Moreover, in our application, statistical tests are highly corrected and classical correction methods would tend to be too conservative. Hence, a pragmatic approach would then tend to avoid selecting a model showing p-values systematically below a given cutoff, for example 5%.

### Model validation

The candidate model is then built based on the entire training dataset using the selected strategy to build the prognostic model. The performance indicators are estimated on the independent test dataset based on the prognostic model predictions. Confidence intervals of the performance indicators can be obtained using bootstrap.

### Implementation

The strategy described in the manuscript was implemented with the following R packages: survival, pec, survivalROC, mfp, rms, MASS, glmnet, penalized.

## Results and discussion

The objective of the application was to build a prognostic model of mortality in a multi-centric cohort of 251 septic shock patients in intensive care units. We considered 3 candidate clinical covariates: the Sequential Organ Failure Assessment Score (C1) which quantifies the “number and severity of failed organs” [33], the lactic acid level (C2), and the white blood cell count (C3) and 6 candidate genes (G1, G2, G3, G4, G5, G6) whose expression levels were measured by RT-qPCR. All of the clinical covariates and gene expressions were measured at the onset of septic shock. Blood samples were collected using PAXgene blood RNA tubes (PreAnalytix, Hilden, Germany). Total RNA was extracted from whole blood using PAXgene™ Blood RNA System Kit (PreAnalytix, Hilden, Germany). Before RNA elution, the residual genomic DNA was digested using the Rnase-Free Dnase set (Qiagen, Hilden, Germany). Extracted RNA was reverse-transcribed into cDNA using SuperScript®;VILO™ cDNA Synthesis Kit (Life Technologies, Chicago, IL). Finally, the PCR was performed on a LightCycler instrument using the standard Taqman Fast Advanced Master Mix PCR kit according to the manufacturer’s instructions (Roche Molecular Biochemicals, Basel, Switzerland). PCR was performed with an initial denaturation step of 10 min at 95^∘^C, followed by 45 cycles of a touchdown PCR protocol (10 sec at 95^∘^C, 29 sec annealing at 68^∘^C, and 1 sec extension at 72^∘^C). The crossing point estimated by the LightCycler software was used as the unnormalized gene expression measure for each gene/sample combination. The gene expression data were normalized using a single reference gene. Although it is recommended to use more than one reference gene [18], we were not able to find more than a single stable gene using the mixed model proposed by Dai et al. [34]. The normalization step was not included in the cross-validation procedure because the normalization method was performed on a sample-by-sample basis. All the candidate covariates were continuous however, according to expert’s recommendations, C2 and C3 have also been treated as binary candidate covariates (denoted by C2 −Fac and C3 −Fac). All the samples were processed across 23 PCR batches. The outcomes were censored at day 14 to avoid considering mortality events not related to the initial septic shock and to avoid departure from the proportional hazard assumption in the Cox model. The Kaplan-Meier estimated survival at day 7 was 0.79 [0.74; 0.84], and Kaplan-Meier estimated survival at day 14 was 0.72 [0.66;0.77]. The training dataset included 156 patients and 44 death events (28%), with samples processed over 14 PCR batches. The test dataset included 95 patients and 27 death events (28%), with samples processed over 9 PCR batches. Unfortunately, the number of events was not balanced across batches as the mean and inter-quartile range for the proportion of events across batches in the training dataset were 0.23 [0.15;0.30] and 0.26 [0.18;0.31], respectively.

**Figure 1:**
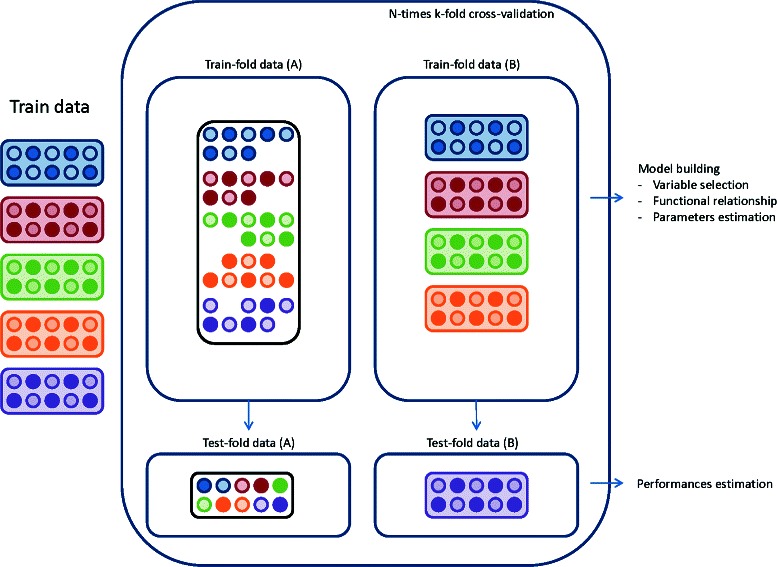
**Patient level (A) vs PCR bacth level (B) resampling strategies.** The training dataset includes 5 batches (on the left of the figure). The figure presents an example of patients resampling in a given fold, and a given iteration. In each batch, gene expression of survivor (open circles) and non-survivor (plain circles) patients are measured. In strategy A, samples are randomly drawn within batches to be included in the training fold-data. In strategy B, entire batches are selected and included in the training-fold data. The model building step is performed on the training-fold data and model performance are estimated on the test-fold data.

As illustrated in Figure 1, two resampling strategies were evaluated: A/20 −times 5 −fold stratified cross-validation with test-fold samples drawn randomly in the training dataset, but ensuring an equal number of events in each test-fold dataset; B/20 −times 5 −fold cross-validation with samples from entire PCR batches drawn randomly to be included in the test fold data. Contrary to strategy B, strategy A does not take into account the within training sample technical variability due to the batch processing. However, with strategy B, given the unbalanced distribution of death events across batches, the outcome distributions are different between and across corresponding training and test −folds.

Table 1 presents the list of prognostic survival model building strategies tested here, i.e. the combination of variable selection methods, functional relationship modeling, survival model, and coefficient shrinkage strategies. These different strategies were chosen because of their ability to accommodate with current prognostic modeling problem as well as to represent the various methodological options available to date in this area.

**Figure 2:**
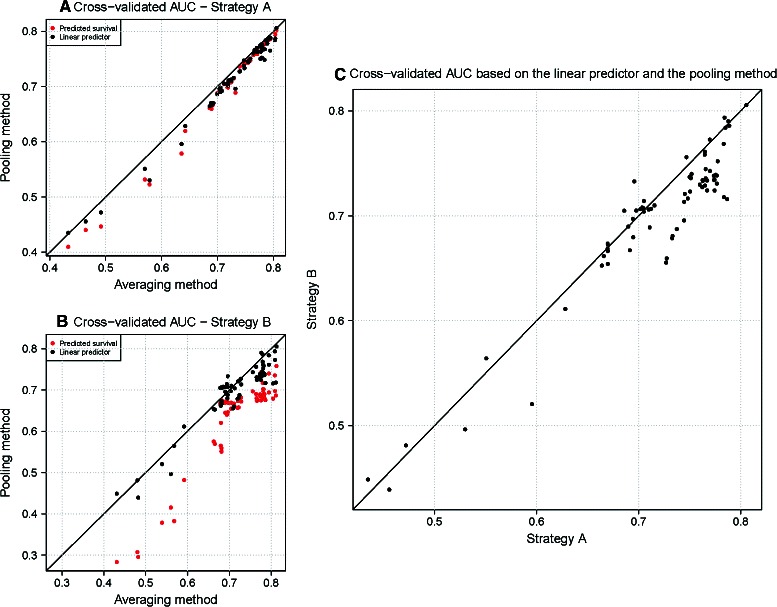
**For some models, resampling strategy A (patient level sampling) tends to over-estimate model performance, compared to sampling strategy B (PCR batch level sampling).** Panel **A** presents the cross-validated AUC at day 7 for all the prognostic models, using resampling strategy A. The cross-validated AUC is estimated using the pooling method (y-axis) and the averaging method (x-axis); red dots correspond to AUC estimations based on the predicted survival and black dots to AUC estimations based on the linear predictor (see Methods section). Panel **B** presents the cross-validated AUC at day 7 for all the prognostic models, using resampling strategy B. Finally, panel **C** compares the cross-validated AUC estimated with strategy A (x-axis) vs. strategy B (y-axis), using the pooling method based on the linear predictor.

First, we compared the impact of the resampling strategies on the performance indicator estimation obtained with the pooling and the averaging methods. Figure 2A presents the cross-validated AUC at day 7 for resampling strategy A where the x-axis corresponds to the AUC estimated using the averaging method based on the predicted survival (note that we would have obtained the same estimations using the linear predictor of the Cox model), and the y-axis corresponds to the AUC estimated with the pooling method using the linear predictor (black dots), and the predicted survival (red dots). For both methods, reported estimations were averaged over the 20 repetitions. For all the models, AUC estimations seemed to be almost equal for both methods, the differences being larger for survival based estimations. Figure 2B presents the same results using resampling strategy B. In this latter case, estimations obtained with the pooling method based on the predicted survival are biased downward. This is due to the fact that the number of events is not the same across batches and, consequently, the predicted survival for the test samples are not comparable across batches and cannot be pooled to estimate the AUC at the end of the cross-validation process. Finally, Figure 2C compares the cross-validated AUC estimated with the pooling method using the linear predictor, for strategy B (y-axis) and strategy A (x −axis). For some models, strategy A led to larger AUC estimates. Indeed, performance estimated with strategy A could be over-estimated because the between PCR batch variability was not properly taken into account. Subsequent results are then reported under strategy B, with the pooling method based on the linear predictor only.

**Figure 3:**
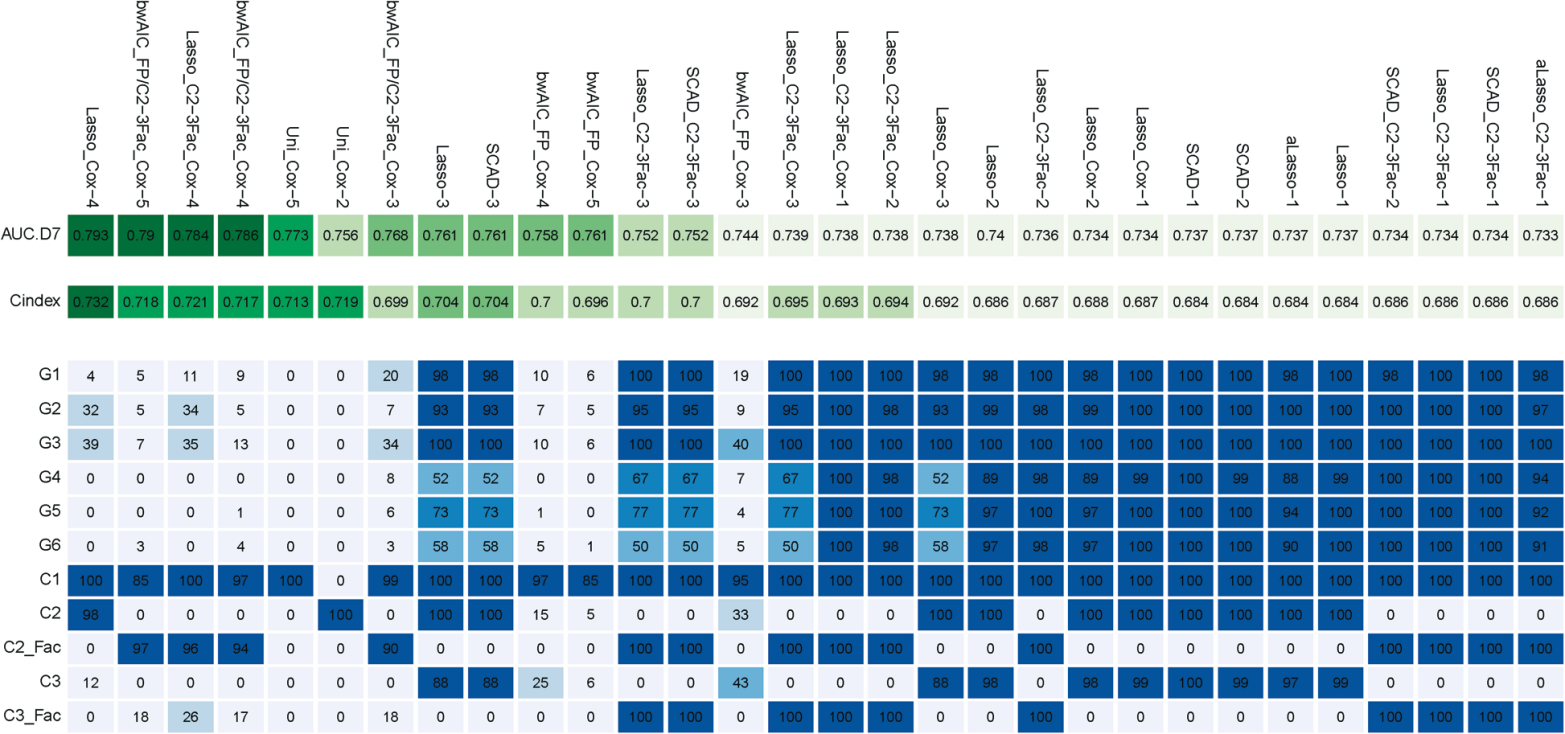
**Performances of the top 30 models obtained with strategy B (PCR batch level sampling).** Each column corresponds to a prognostic survival model. The first 2 rows report respectively the cross-validated AUC at day 7 and the cross-validated C-index (based on the linear predictor). Darker colors correspond to better performances. The 11 next rows correspond to each candidate covariate (G1 to G6, C1, C2, C3 and C2_Fac and C3_Fac when the clinical covariates 2 and 3 have been dichotomized according to clinical expert knowledge). The number within each cell gives the percentage of selection of each variable in each model across the cross-validation iterations. Darker colors correspond to a higher selection frequency.

Figure 3 presents the performance of the top 30 models obtained with resampling strategy B (top panel) together with the frequency of selection of candidate covariates across the cross −validation iterations (bottom panel). As we can see, the top 30 models showed highly similar performances. Among these top 30 models, 22 used penalized approaches for variable selection (lasso, adaptive lasso, and SCAD). As described in Table 1, penalized model names were suffixed with 1, 2, 3, 4, or 5; the larger the suffix, the more sparse (less covariates selected) the model. In general, models suffixed by 1 or 2 selected all the covariates, models suffixed by 3 selected the 3 clinical covariates and most of the genes. The models suffixed by 4 were more parsimonious and selected only 2 or 3 clinical covariates. Interestingly, penalized approaches which are traditionally used in high dimensional settings, performed well here in a *p*<*n* setting. The list of the best models could be drastically shortened retaining a single strategy per variable selection method or level of penalization for penalized approaches (i.e. a single model per level of complexity in terms of number of covariates included): e.g. the Lasso_Cox-4, the Uni_Cox-5, the Lasso-3, and the Lasso_C2-3Fac_Cox1 models. Indeed, retaining very close models, for example bwAIC_FP/C2-3Fac_Cox-5 and Lasso_C2-3Fac_Cox-4 was of little interest, because these two models always selected the same covariates and the model coefficients were very close.

**Figure 4:**
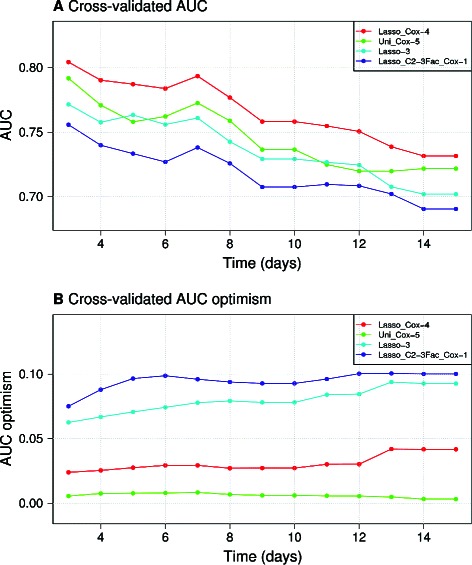
**The Lasso_Cox −4 model offers the best compromise between performance and validation surprise.** Panel **A** presents the cross-validated time-dependent AUC for the 4 candidate models using strategy B (PCR batch level sampling). Panel **B** the cross-validated using strategy B time-dependent “validation surprise” computed from the AUC for the 4 candidate models.

In order to select the best out of four candidate models, we then looked at additional the performance indicators previously introduced. Figure 4 panel A and B present the cross-validated time-dependent AUC and the associated optimism for the four candidate models, respectively. The Lasso_Cox-4 model exhibited the best performance and the smallest optimism, which may be related to the fact that it was the most parsimonious among the four models.

**Figure 5:**
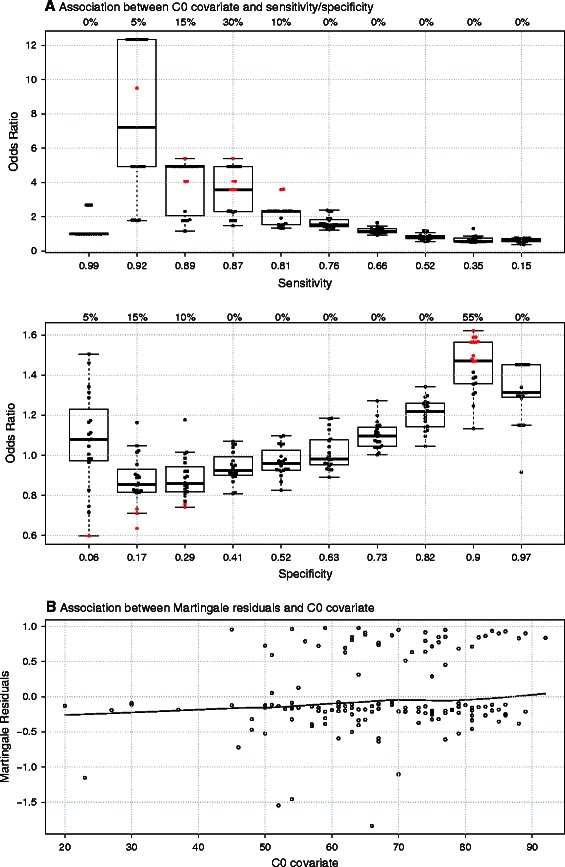
**Omitted covariate is associated with time-dependent sensitivity and specificity (Panel A) and Martingale residuals (Panel B) in the selected model (Lasso_Cox −4).** Panel **A** presents the association between the C0 omitted covariate and the time-dependent sensitivity and specificity at day 7. Each boxplot corresponds to the odds ratio (OR) across all the cross-validation iterations, for a given cut-off on the linear predictor of the model (i.e. a given combination of sensitivity and specificity). Red points correspond to OR with p-values <0.05. The number above the boxplots gives the proportion of p-values <0.05 and the numbers below the boxplot, the sensitivity and the specificity values for a given cut-off on the linear predictor. Panel **B** presents the scatter plot of the Martingale residuals and C0 covariate.

Based on this observation, we thus decided to select the Lasso_Cox-4 model as the best candidate and checked that its performance did not depend on omitted clinical covariates. For illustration purpose, we present the impact of a potentially omitted clinical covariate C0 (age of the patient) on the model performance. This clinical covariate was omitted because we had to limit the number of candidate covariates to respect the “5 to 10 EPV” rule for the training set sample size. First, Martingale residuals of the Lasso_Cox-4 model were plotted against the omitted C0 covariate and, a loess smoothing spline was added to better evaluate the degree of residual survival association with the C0 covariate. As can be seen in Figure 5B, the C0 covariate seemed to be slightly associated with the Martingale residuals, indicating that the model fit could be improved by adding the C0 covariate and that the model performance may differ for different C0 values. To further explore this effect, Figure 5A presents the association between time-dependent sensitivity and specificity at day 7 and the C0 covariate. In our application the vital status was known for all the patients at day 7: they fell into two categories, already deceased or still alive; hence there was no difficulty to fit a logistic model to test the effect of the omitted covariate on the sensitivity and specificity. In Figure 5A, each boxplot corresponds to the odds ratio of association for all the cross-validation repetitions for a given *c* value cut-off on the linear predictor of the Lasso_Cox-4 (red points indicating OR with p-value below 5%). As can be seen in Figure 5A, the C0 covariate was slightly associated with very high sensitivity leading to larger sensitivity for people with larger C0 values, while the prognostic model seems to present a higher specificity for people with larger C0 values, although not significant for most odd-ratios. The same approach was used for other covariates and no evidence of association was found for Lasso_Cox-4 model. Thus, we confirmed that this strategy is the best one here and can be retained for the subsequent validation step. Again, note that the p-values should be interpreted with caution due to the large number of test performed.

Finally, the entire training dataset was used to build the prognostic model. The final Lasso_Cox-4 model included the following covariates: C1: HR =1.14(*p*=0.01), C2: HR =1.16(*p*=0.0002), G2: HR =0.62(*p*=0.06), and G3: HR =1.64(*p*=0.02). Although, not often selected in the cross-validation process, G2 and G3 were selected on the whole training dataset.

**Figure 6:**
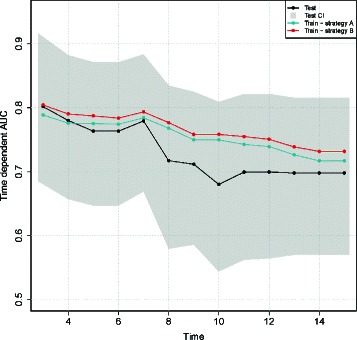
**The validation surprise observed with strategy B (PCR batch level sampling) is smaller than the validation surprise observed with strategy A (patient level sampling).** Strategy A cross-validated time-dependent AUC (blue); strategy B cross-validated time-dependent AUC (red); validated time-dependent AUC on the test dataset (black) and bootstrap confidence intervals (grey polygon) (95% of the boostrap samples distribution) for the Lasso_Cox −4 model.

Figure 6 compares the cross-validated performances estimated on the training dataset using strategy A (blue line), strategy B (red line) and validated performances on the test dataset (black line), for the Lasso_Cox-4 selected model. The cross-validated time-dependent AUC estimated with strategy A and B were comparable. The validated AUC on the test dataset decreased more importantly after day 7, than the cross-validated estimations, although the training cross-validated AUC fall within the confidence intervals of the test dataset validated AUC.

### Discussion

In this article, we presented and illustrated a complete process to train, select and validate a biomarker prognostic model based on RT-qPCR gene expression and clinical covariates. We discussed two resampling strategies: A) a stratified cross-validation resampling strategy, where test-fold samples were randomly sampled within batches but ensuring that the number of events was the same across folds and B) a cross-validation strategy that takes into account the study design and the fact that samples were processed by batch. In this strategy, entire batches were set apart in the test-fold data to capture the impact of the technical and biological variability on performance estimation. An expected benefit of the resampling strategy B, was the selection of markers less sensitive to the batch-to-batch variability, leading to better generalization. Indeed, training and test samples were processed in different batches and it was interesting to capture the potential validation surprise during the cross-validation process due to batch sample processing. In our example, we found that the cross-validated AUC was larger when estimated with strategy A than with strategy B for some models. As a recommendation, we suggest carrying out both resampling strategies and choosing the most conservative, cross-validated AUC estimation scheme for model selection. It is interesting to evaluate the impact of the resampling strategy on the performance estimation. Unfortunately, strategy B could lead to a test-fold data outcome distribution that is very different from training-fold data outcome distribution when batches are unbalanced with respect to the outcome. In this case, it is not possible to estimate overall performance indicators based on the predicted survival (such as the Brier score), because mean survival in the training and test-fold data would be too different. On the contrary, discrimination performance indicators (eg. C-index, AUC), can be estimated without bias, using the pooling or averaging method, when defined on predicted scores insensitive to differences between training-fold and test-fold outcome distribution. For example, the linear predictor of a Cox model, that treats the baseline hazard as a nuisance parameter can be used as a predictive score from which to derive the performance indicator.

The bootstrap is also another well-established resampling strategy. For survival data, Gerds and Schumacher [9] have derived an analogue of the 0.632+ bootstrap error estimator introduced by Efron and Tibshirani [35]. This approach could indeed be preferred over cross-validation which is known to yield upwardly biased error estimates since only a subset of the training set is used to train the model. Nevertheless, it is not straightforward to extend the batch sampling strategy to the bootstrap procedure as the 0.632+ estimator will no longer hold since here the probability that an observation belongs to a given bootstrap sample is no longer 0.632.

Although cross-validated performance was the dominant criterion for model selection, we used additional criteria to weigh the estimated prediction performance at the expense of other risks of generalization failure. The proposed criteria are *i)* the stability of the selected variables across cross-validation repetitions, *ii)* the clinical interest in the selected variables, *iii)* the model optimism, and *iv)* the impact of the omitted variables on the model performance.

A natural extension of our work is to build a prognostic index that would make the selected biomarker model more useful clinically. This prognostic index will facilitate the clinical interpretation of the model outcome by stratifying patients into a small number of risk categories: for instance, distinguishing low-risk, intermediate and high-risk patients. One possibility is to create the prognostic index by defining cutoffs on the linear predictor of the selected and validated prognostic biomarker model in order to define the desired risk categories. We recommend estimating the cutoffs by a cross-validation process similar to the one used to select the prognostic model as we believe this strategy yields better results than the one based on defining cutoffs for each individual marker and then combining them to create ad-hoc prognostic groups.

In case of predictor variables with missing values, it is possible to perform Multiple Imputations (MI). The idea of MI is to create *m* “completed datasets” with filled-in missing values. The imputation model for the missing values should contain all the candidate predictors, all the covariates that could be associated with the covariates with missing values, the event status and the survival time [36]. The analysis is then performed on the *m* completed datasets using standard tools, and the results are combined into one final estimate using the Rubin’s rule [37]. In the context of prognostic model building, two difficulties remain: how to perform variable selection and how to estimate model performance based on the imputed datasets? Wood et al. [38] compared different approaches to perform backward stepwise variable selection, and concluded that the only method that preserved the type I error was to select covariates based on the Rubin’s rule estimated p-values at each step of the backward stepwise procedure. Musoro et al. [39] presented a procedure based on bootstrap resampling and multiple imputations to perform both variables selection via lasso and estimation of prognostic model performance. The authors recommend incorporating the MI procedure within the resampling step, i.e. to draw *m* completed datasets for each bootstrap sample. Briefly, for a given bootstrap sample, lasso estimates are averaged over all the imputed datasets to build a final model (meaning that zero and non-zero coefficients are averaged). Validated performance is then estimated on imputed datasets including all the samples (not only the bootstrap samples). Using this resampling framework in simulations, the internal estimated optimism was the closest to the external estimated optimism. An alternative approach is proposed by Chen et al. [40] who performed group-lasso to consistently select covariates across imputed datasets.

Finally, additional future work would be needed to validate our strategy in high-dimensional settings (*p*>>*n*), in particular for microarray based prediction model building. In microarray studies, samples are also processed by batch, and it is also relevant to set apart entire batches in test-fold data. Of course, some statistical aspects may change to accommodate the specificity of the high-dimensional setting (e.g. the sizing strategy of the training dataset, see for instance [15]). As for the present RT-qPCR study, an evaluation based on real datasets will be required to validate our approach on other transcriptomic platforms.

## Conclusions

In conclusion, we presented resampling methods in order to estimate the performance of RT-qPCR prognostic models on a training dataset. These methods are expected to prevent any upward biases due to unaccounted technical and biological variability that may arise from the experimental and intrinsic design of the RT-qPCR assay.

Although the estimation of the performance of the candidate models is a pivotal indicator to help select the best model, the final decision of selecting one or a few models requires consideration of additional criteria. We propose to use the stability of the selected variables, the model optimism, and the impact of the omitted variables on the model performance. These criteria should be weighted in accordance with the objective of the study.

Model building strategies developed in high-dimensional settings can also be very efficient in lowdimensional settings, in particular for covariate selection and coefficient penalization. In our example dataset with more individuals than variables, penalized survival models yielded the highest performances.

## Additional file

Additional file 1
**Impact of the survival distribution differences between training and test-fold data on the C-index estimation.** Additional file 1 is a.pdf file with simulations results on the impact of the survival distribution differences between folds on the C-index estimation.
