# Supplementary material for: A strategy to build and validate a prognostic biomarker model based on RT-qPCR gene expression and clinical covariates
Source: BMC Bioinformatics. 2015 Mar 28;16:106. doi: 10.1186/s12859-015-0537-9 (PMC4384357; doi:10.1186/s12859-015-0537-9)

Additional file 1 - Impact of the survival distribution differences between training and test-fold data on the C-index estimation

We performed simulations to illustrate why the linear predictor of the Cox model should be preferred to the estimated survival for the C-index estimation.

First, we simulated a dataset with a unique covariate not associated with the survival and second a dataset with a unique covariate associated with the survival.

1 No association between the covariate and the survival time

First, we simulated a dataset with 1000 patients with a unique predictor X not associated with the survival: X followed a standard Normal distribution, and the uncensored survival time T followed a Weibull distribution with shape equal to 1, and scale equal to 10. In this setting, X and T were independent and no censoring was added. In this case, the C-index was expected to be close to 0.5 because the X covariate was not a survival time predictor.

We considered 5 folds, and the patients were sampled within folds according their survival time in order to study the impact of an outcome unbalanced between folds (e.g. strategy B with the outcome unbalanced between batches). As can be seen in figure 1, the survival distributions were very different across test-folds. Since the covariate was not associated to the survival time, the covariate distribution did not vary across test-folds.

A Cox model $h(t) = h_0(t)\exp(-X\beta)$ was trained in each fold; as expected all the estimated $\hat{\beta}$ coefficients were close to 0: $\hat{\beta}_{\text{train}-\text{fold}-1} = 0.04$, : $\hat{\beta}_{\text{train}-\text{fold}-2} = 0.05$, $\hat{\beta}_{\text{train}-\text{fold}-3} = 0.07$, $\hat{\beta}_{\text{train}-\text{fold}-4} = 0.06$, and $\hat{\beta}_{\text{train}-\text{fold}-5} = 0.04$.

Figure 2 presents the baseline estimated hazard in each training-fold. As expected, the baseline estimated hazards were very different between folds. For example, the baseline hazard was larger in training-fold 1, because the samples from test-fold 1, with the largest survival times (see figure 1) were not used for this estimation.

Then, the C-index was estimated with the pooling method from the linear predictor: C-index= 0.51 and from the survival estimated at time 5; C-index=0.30. The C-index estimated from the linear predictor was almost equal to the expected C-index=0.5, whereas the C-index estimated from the predicted survival at time 5 was biased downward. Indeed, in the latter case, the test-fold patients were ordered according to the training-fold estimated hazard which was very different across folds. Hence, the predicted survival at time 5 could not be compared between folds and should not be used to estimate the C-index with the pooling method (see figure 3).

Figure 1: Left panel: X-covariate distribution across test-folds; Right panel: Survival time distribution across test-folds

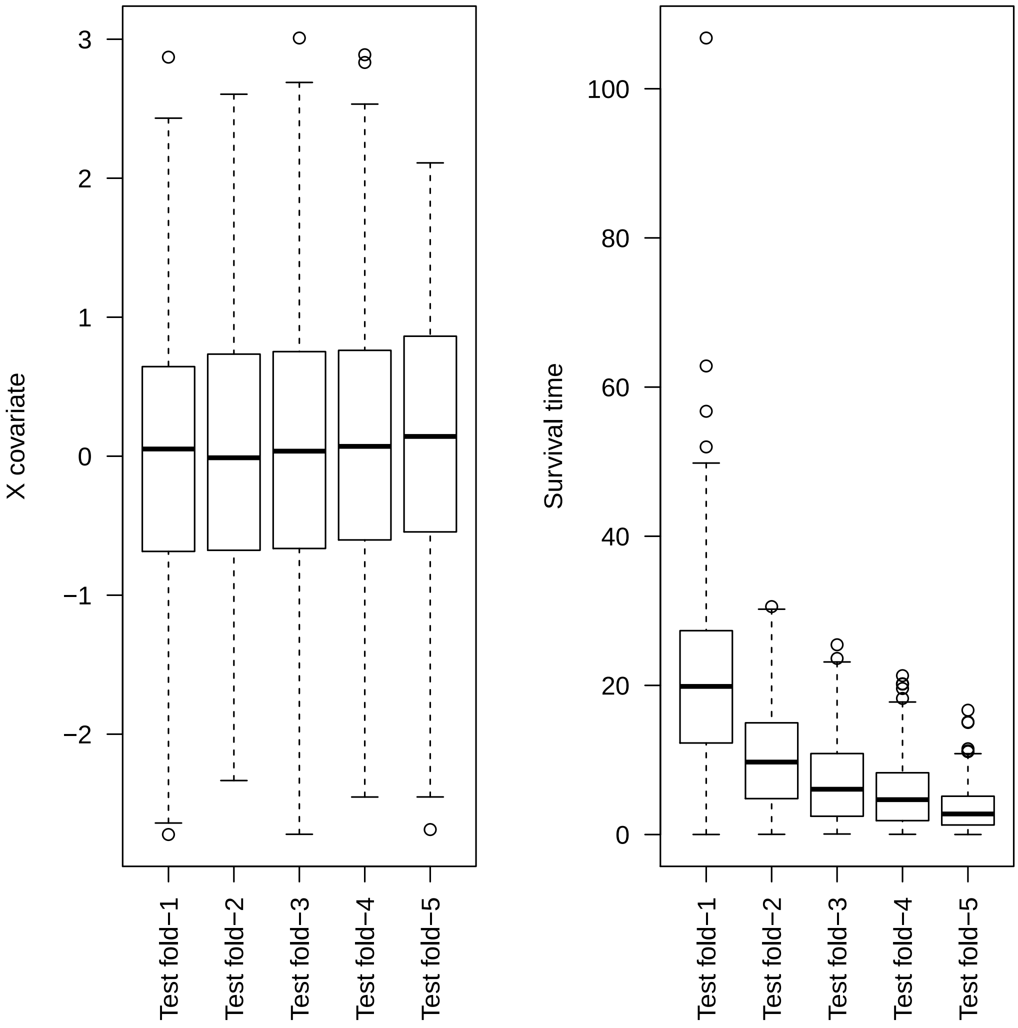

Figure 2: Baseline hazard function in each training-fold

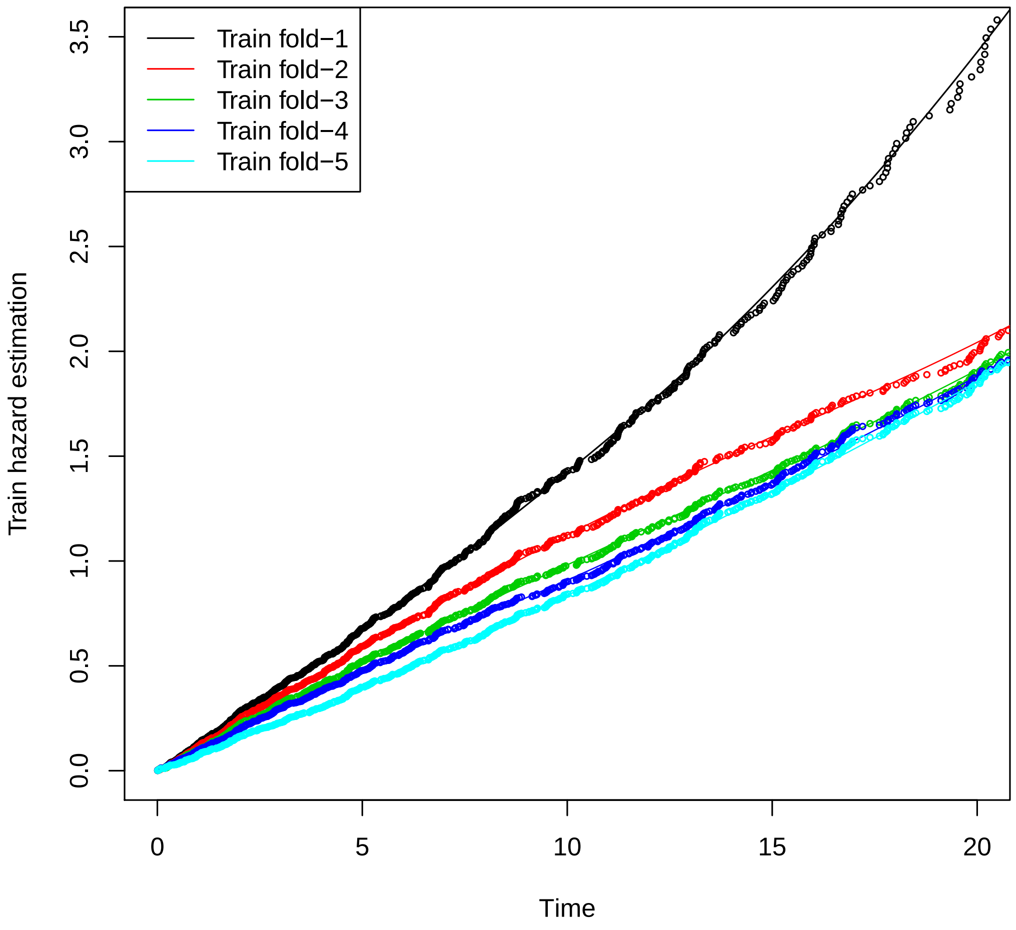

Figure 3: Left panel: Predicted linear predictor across test-fold ; Right panel: Predicted survival at time 5 across test-fold

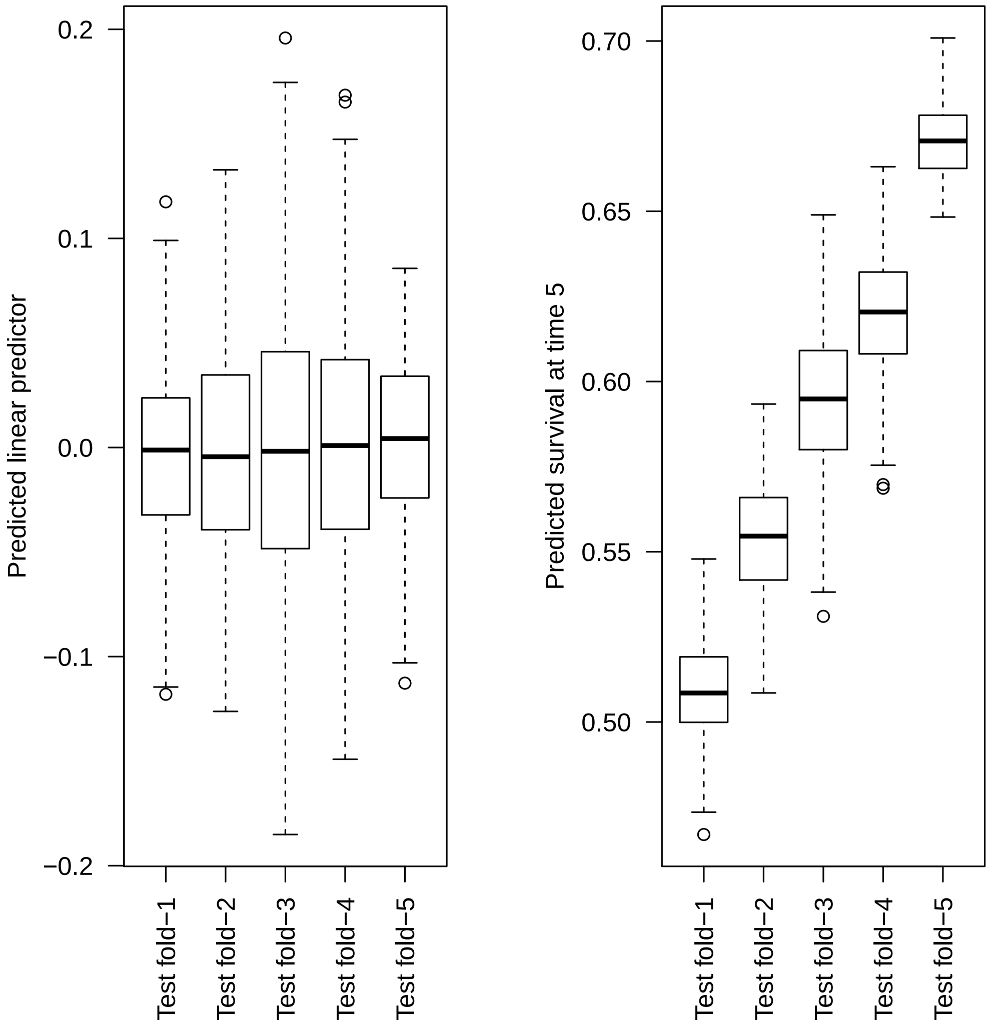

2 Association between the covariate and the survival time

Here, we simulated a dataset with 1000 patients with a unique predictor X associated with the survival: X followed a standard Normal distribution, and the uncensored survival time T followed a Weibull distribution with shape equal to 1, and scale equal to $\exp(X\beta)$, with $\beta = 1$. This formulation of the Weibull model assumes a proportional effect of the covariate X on the hazard, with hazard ratio equal to $\exp(1)$. No censoring was added. In this case, the C-index was expected to be larger than 0.5, because the X covariate was associated to the survival.

As previously, we considered 5 folds, and the patients were sampled within folds according their survival time. As can be seen in figure 4, both the covariate and the survival distributions were very different across folds, because the covariate was associated with the survival time and consequently to the fold index.

Figure 4: Left panel: X-covariate distribution across test-folds; Right panel: Survival time distribution across test-folds

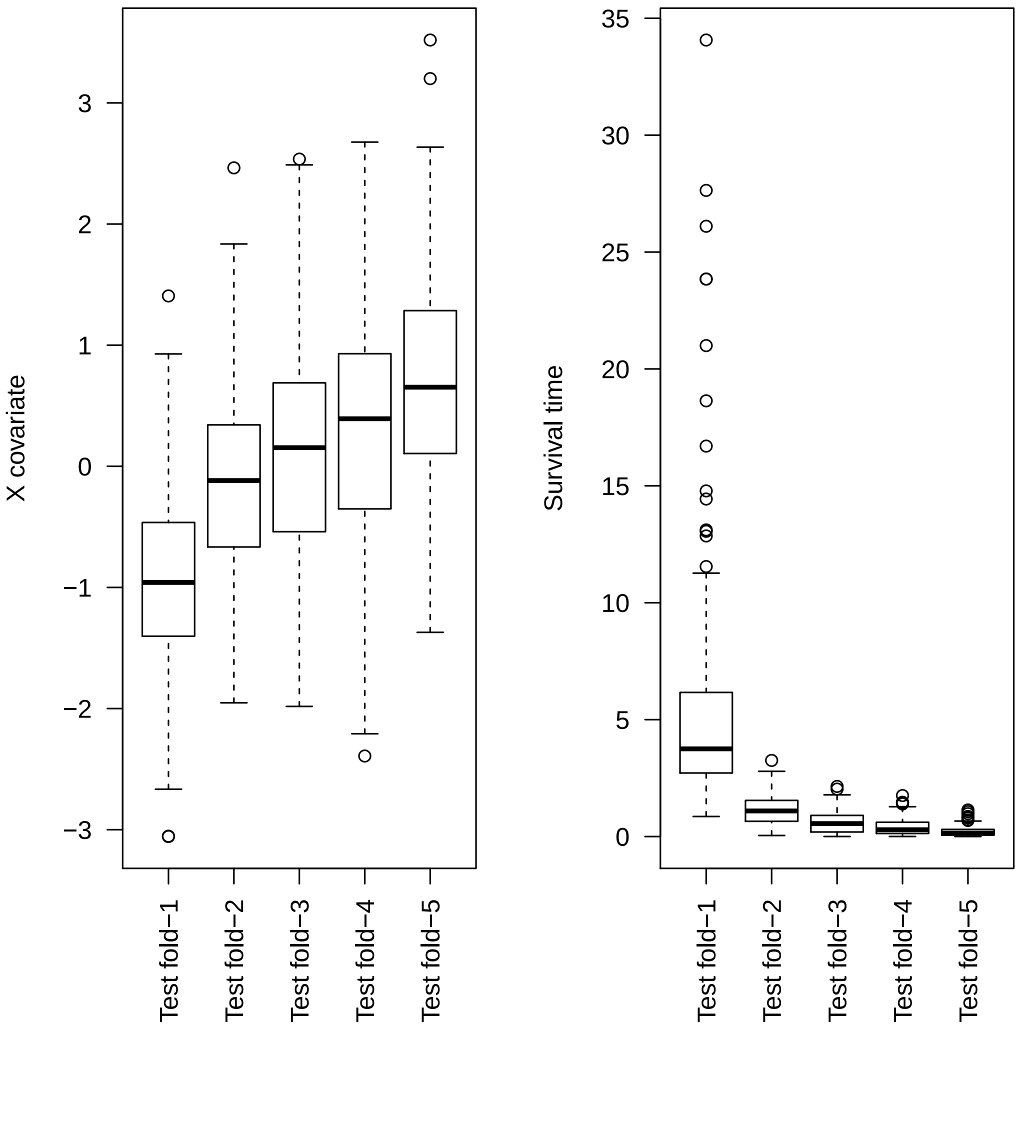

A Cox model $h(t) = h_0(t)\exp(-X\beta)$ was trained in each fold; the estimated $\hat{\beta}$ coefficients were $\hat{\beta}_{\text{train-fold-1}} = 0.62$, $\hat{\beta}_{\text{train-fold-2}} = 0.96$, $\hat{\beta}_{\text{train-fold-3}} = 1.01$, $\hat{\beta}_{\text{train-fold-4}} = 1.03$, and $\hat{\beta}_{\text{train-fold-5}} = 0.95$. As can be seen above, the β coefficient estimated in the first training-fold was biased; this can be explained by the fact that the survival distribution in the test-fold 1 was very different from the survival distribution in the other test folds.

Figure 5 presents the baseline estimated hazard in each training-fold. As expected, the baseline estimated hazards were very different between folds. For example, the baseline hazard was larger in training-fold 1, because the samples from test-fold 1, with the largest survival times (see figure 4) were not used for this estimation.

Figure 5: Baseline hazard function in each training-fold

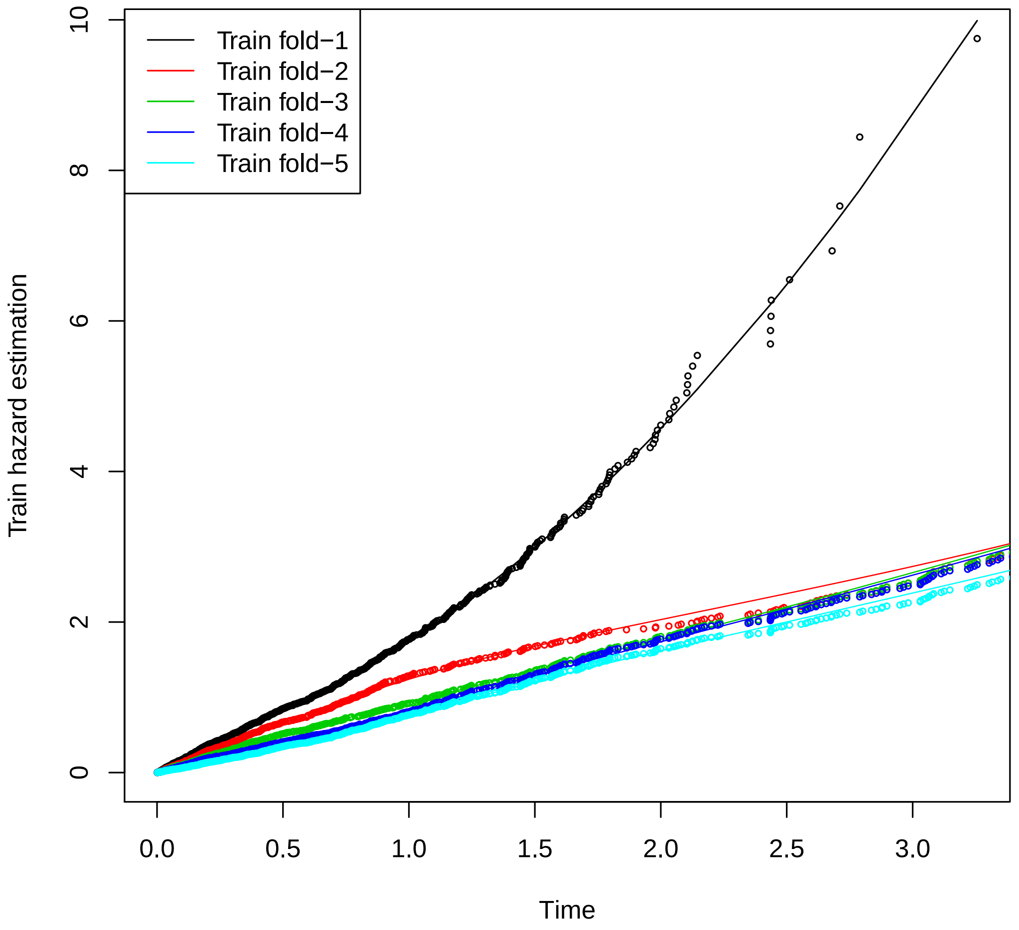

Then, the C-index was estimated with the pooling method from the linear predictor: C-index= 0.72 and from the survival estimated at time 1; C-index=0.63. The apparent C-index estimated on the whole dataset was 0.71. The C-index estimated from the linear predictor was almost equal to the apparent C-index=0.72, whereas the C-index estimated from the predicted survival at time 1 was biased downward. When estimated from the linear predictor, the C-index estimation bias only depends on the $\hat{\beta}$ coefficients estimation bias, whereas when estimated from the predicted survival, the C-index estimation bias depends both on the $\hat{\beta}$ coefficients estimation bias and the difference between the baseline hazards estimated in the training-folds. Hence, the predicted survival at time 1 could not be compared between folds and should not be used to estimate the C-index with the pooling method.

Figure 6: Left panel: Predicted linear predictor across test-fold ; Right panel: Predicted survival at time 1 across test-fold

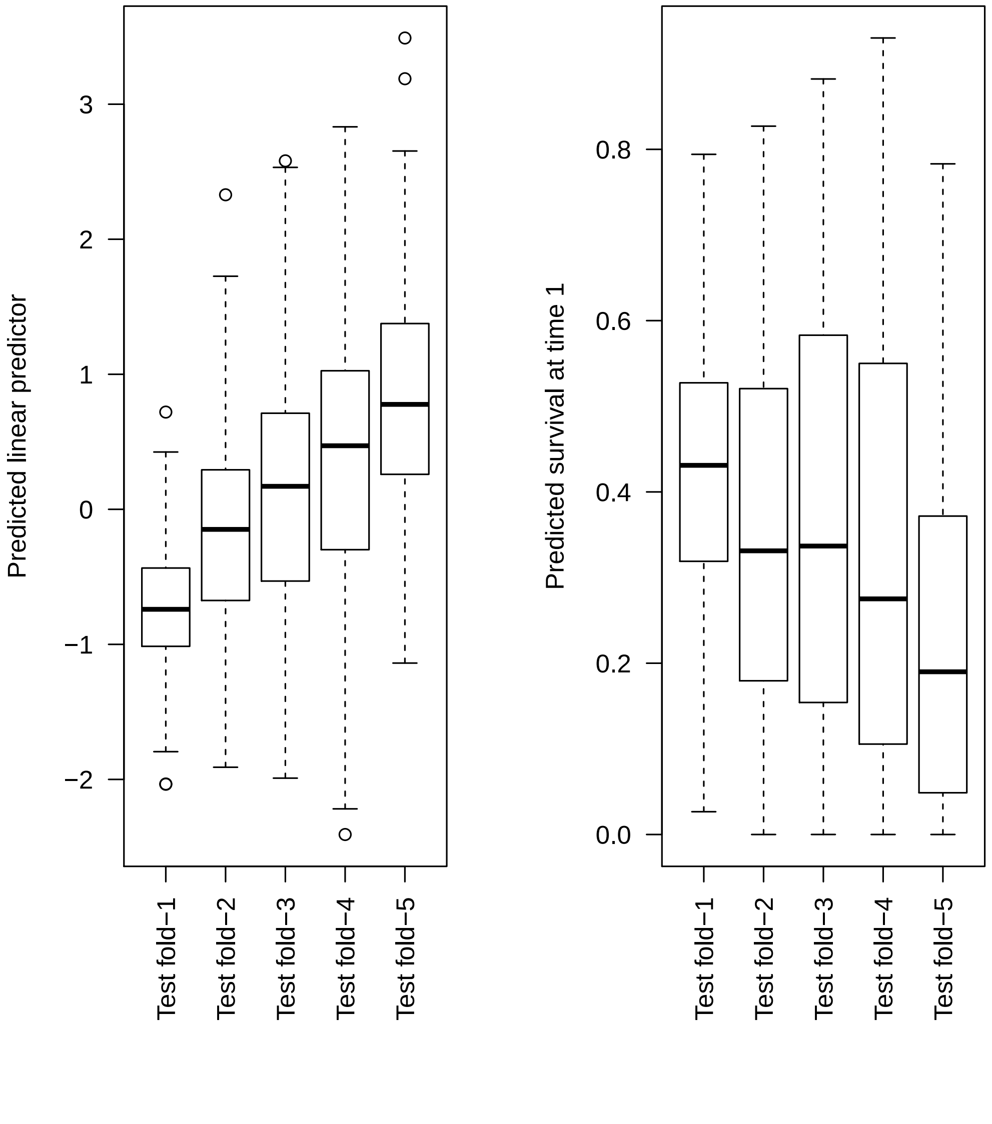

Supplement: Additional file 1 — Impact of the survival distribution differences between training and test-fold data on the C-index estimation. Additional file 1 is a.pdf file with simulations results on the impact of the survival distribution differences between folds on the C-index estimation. [file 12859_2015_537_MOESM1_ESM.pdf]
